# Fulfilling outpatient medicine responsibilities during internal medicine residency: a quantitative study of housestaff participation with between visit tasks

**DOI:** 10.1186/s12909-016-0665-6

**Published:** 2016-05-10

**Authors:** Jason Hom, Ilana Richman, Jonathan H. Chen, Baldeep Singh, Casey Crump, Jeffrey Chi

**Affiliations:** Department of Medicine, Stanford University School of Medicine, 300 Pasteur Drive, HC007, Stanford, CA 94305-5133 USA; Center for Innovation to Implementation at the Veteran Affairs Palo Alto Health Care System, Palo Alto, CA USA; Center for Health Policy/Primary Care and Outcomes Research at Stanford University, Stanford, CA USA

**Keywords:** Internal medicine residency, Continuity clinic, Duty hours

## Abstract

**Background:**

Internal Medicine residents experience conflict between inpatient and outpatient medicine responsibilities. Outpatient “between visit” responsibilities such as reviewing lab and imaging data, responding to medication refill requests and replying to patient inquiries compete for time and attention with inpatient duties. By examining Electronic Health Record (EHR) audits, our study quantitatively describes this balance between competing responsibilities, focusing on housestaff participation with “between visit” outpatient responsibilities.

**Methods:**

We examined EHR log-in data from 2012–2013 for 41 residents (R1 to R3) assigned to a large academic center’s continuity clinic. From the EHR log-in data, we examined housestaff compliance with “between visit” tasks, based on official clinic standards.

We used generalized estimating equations to evaluate housestaff compliance with between visit tasks and amount of time spent on tasks. We examined the relationship between compliance with between visit tasks and resident year of training, rotation type (elective or required) and interest in primary care.

**Results:**

Housestaff compliance with logging in to complete “between visit” tasks varied significantly depending on rotation, with overall compliance of 45 % during core inpatient rotations compared to 68 % during electives (*p* = 0.01). Compliance did not significantly vary by interest in primary care or training level. Once logged in, housestaff spent a mean 53 min per week logged in while on electives, compared to 55 min on required rotations (*p* = 0.90).

**Conclusions:**

Our study quantitatively highlights the difficulty of attending to outpatient responsibilities during busy core inpatient rotations, which comprise the bulk of residency at our institution and at others. Our results reinforce the need to continue development and study of innovative systems for coverage of “between visit” responsibilities, including shared coverage models among multiple residents and shared coverage models between residents and clinic attendings, both of which require a balance between clinic efficiency and resident ownership, autonomy and learning.

## Background

Internal Medicine (IM) residency programs are designed to provide trainees with comprehensive training which encompasses a combination of inpatient and outpatient experiences in general medicine and medical sub-specialties [1, 2]. Residency graduates can then pursue further subspecialty training through fellowships or pursue careers as hospitalists or primary care providers. While IM residency programs throughout the country adhere to this guiding principle, the actual balance between inpatient and outpatient training is program dependent. In fact, most IM residency programs are weighted toward time-intensive inpatient rotations rather than outpatient general medicine clinic [2, 3]; consequently, achieving optimal continuity of care with a panel of outpatients can be difficult, particularly in this era of duty hour reform. The majority of residents and program directors find difficulties with competing inpatient-outpatient responsibilities [4], which may lead to IM trainees feeling less prepared to address outpatient issues compared to inpatient issues, particularly compared to their colleagues in family medicine [3, 5].

In ambulatory practice, patient visits are marked by relative brevity and are often irregularly scheduled, with continuous relationships between patients and doctors developing gradually over time [6]. Consequently, the ability for residents to communicate with patients between visits is a key component to their continuity experience. Indeed, the Alliance for Academic Internal Medicine (AAIM) Education Redesign Task Force highlighted the need for reform to “improve ambulatory training by providing patient-centered longitudinal care that addresses the conflict between inpatient and outpatient responsibilities” [1].

Although there has been increasing attention toward various continuity clinic scheduling models in recent years [7], attention to outpatient responsibilities outside of actual continuity clinic hours has historically received relatively little attention. Recently, some investigators have argued that “a more encompassing definition of continuity” should include “not only visit continuity, but also lab follow-up and phone triage encounters” [8], given the importance of these “between visit” tasks toward achieving AAIM’s goal of “patient-centered longitudinal care” [1]. These investigators found that implementing non-traditional scheduling models, such as 4 + 1 scheduling, allowed housestaff more opportunities to address their patients’ lab results more often. Other investigators have looked at whether self-review or peer-review of charts can increase housestaff performance on outpatient laboratory follow-up; peer-review appeared to be beneficial [9].

To our knowledge, however, no prior study has actually studied how busy inpatient services affect housestaff ability to attend to these “between visit” tasks. We seek to fill a gap in prior knowledge by quantitatively assessing housestaff compliance with individual responsibility for EHR log-ins to complete “between visit” tasks. We use scheduling data to assess for differences in compliance based on whether housestaff were on a core inpatient rotation or elective rotation, and we additionally assess whether housestaff career interest impacts compliance.

## Methods

### Housestaff expectations & responsibilities

During the time of our study, housestaff assigned to Stanford Internal Medicine (SIM) Clinic were scheduled clinic time based on a traditional scheduling model where trainees are excused from their rotation duties in order to attend periodic afternoon outpatient continuity clinic.

The system at Stanford is designed so that interns (who are less efficient when they start) see less patients in clinic initially. Consequently, for the first 3–6 months, interns are scheduled for 3 patients per clinic half day. Subsequently, for the remainder of their intern year and for their R2 and R3 years, housestaff are scheduled for 5 patients per clinic half day. Furthermore, interns are scheduled for approximately 30 clinic half days per year, while R2s and R3s are scheduled for approximately 45 clinic half days per year.

During the study period, housestaff assigned to the SIM Clinic were responsible for reviewing their clinic’s Electronic Health Records (EHR) at least twice per week as a component of their continuity clinic duties. Therefore, compliance in our study is defined as occurring when two or more logins per week were completed, as defined by the clinic regulations. Residents were not aware that this particular study was being conducted. However, as part of their formal clinical expectations from the Stanford Internal Medicine clinic, they had very specific instructions to login at least biweekly to the EMR and check their outpatient inboxes for messages from patients, medication re-fill requests, etc. These instructions were provided both in written orientation materials as well as during clinic orientation sessions.

The outpatient continuity clinic utilizes the EPIC EHR platform (Madison, WI), which allows users to securely login through remote access, with a 20 min automatic log off due to inactivity. Responsibilities typically included, but were not limited to, replying to patient emails, follow-up on patient labs, studies, and imaging, updating patients on results, addressing medication refills and documenting telephone encounters.

### Study population & data collection

The SIM Clinic is the outpatient primary care continuity clinic where 20 interns (R1), 17 second year residents (R2) and 8 third year residents (R3) were assigned during the 2012–2013 academic year.

### Context

The Stanford Internal Medicine Clinic provides primary care to a diverse population of patients from different ethnic and socioeconomic backgrounds. During our study period, there were 14 different attending physicians and 45 residents who worked in the clinic. Each clinic visit between a resident and a patient was supervised by an attending.

### Data collection

Our data collection was approved by the Stanford Institutional Review Board. Using EPIC Clarity Reporting provided by our hospital informatics support team, we generated an audit report describing individual logins and time spent by each trainee during the study period. Each trainee was identified by his or her unique electronic log-in ID. To capture housestaff activity related specifically to “between visit” continuity clinic responsibilities, our study targeted logins that occurred through remote access and only included data from trainees rotating “off-site” on inpatient and sub-specialty consultation services at our affiliated county hospital and veterans hospital, which do not share the same EHR platform. In other words, we targeted instances in which housestaff would be remotely logging in to the EHR platform for their continuity clinic “between visit” responsibilities, either from other hospitals or from home.

Audit logs from weeks where trainees were rotating at our main hospital were excluded due to difficulty in separating rotation tasks from continuity clinic tasks, given the shared EHR. Additionally, trainee rotations were excluded from the data set if they were immediately preceded by an inpatient rotation at the main hospital to preclude activity related to prior rotation responsibilities such as discharge summaries. This data was then matched to a resident scheduling database, allowing us to evaluate login data based on rotation assignment. A spreadsheet containing our raw data is available as a supplementary file entitled De-Identified Data.

For our study, we extracted data regarding resident career interests from surveys that were conducted as part of the residency program’s periodic check-ins with residents.

### Statistics

We used generalized estimating equations (GEE) to evaluate predictors of housestaff adherence to between-visit follow up guidelines. The primary outcome for these models was compliance with the goal of logging into the EMR at least twice weekly. We examined housestaff year, elective or nonelective time, and interest in primary care as predictors of adherence. We also modeled an interaction between elective rotation and training year. For these models, we used GEE with a logit link, exchangeable correlation, and robust standard errors and we expressed results as predicted probabilities rather than odds ratios, for ease of interpretation. We used the Wald test to assess overall group differences (e.g., differences according to residency level of training).

We also examined time spent logged in to the EMR. For these analyses, we again used GEE, this time with mean time spent logged in conditional on logging in at least once as our main outcome. We included year of training and elective rotation in our model, as well as an interaction.

## Results

Of the 45 residents assigned to SIM Clinic, our study population included 41 residents: 20 first year residents, 15 second year residents, and 6 third year residents; four residents (two R2s and two R3s) were excluded because they had no eligible weeks for analysis. Residents in our sample had a median 5 eligible weeks for analysis. Overall, when surveyed, 12 % of residents indicated that they were planning a career in primary care. Sample characteristics are further detailed in Table 1.

**Table 1:** Sample characteristics

Characteristic	Total (*N* = 41)	R1 (*N* = 20)	R2 (*N* = 15)	R3 (*N* = 6)
Total eligible weeks	240	156	50	34
Elective weeks (%)	30 (13)	10 (6)	12 (24)	8 (23)
Median eligible weeks per resident (Interquartile Range)	5 (3–8)	8 (5.5-10.5)	3 (2–4)	5 (3–8)
Career interest				
Primary care (%)	5 (12)	2 (10)	2 (13)	1 (17)
Hospitalist medicine (%)	9 (21)	3 (15)	5 (33)	1 (17)
Medical specialties (%)	27 (67)	15 (75)	8 (53)	4 (67)

R1 = First-year resident (Intern), R2 = Second-year resident (Junior Resident), R3 = Third-year resident (Senior Resident)

Residents were more likely to be compliant with logging into the EMR while on elective rotations (Table 2). After adjusting for year of training and interest in primary care, we found that during electives, residents were compliant 68 % of the time (95 % CI 53–85) compared to 45 % of the time during core inpatient rotations (95 % CI 34–56, 23 percentage point difference, *p* = 0.01). Results were similar in an unadjusted model.

**Table 2:** Compliance with outpatient duties policy by training year and rotation type

	Required rotation % (95 % CI)	Elective % (95 % CI)	Percentage point difference % (95 % CI)	*p*-value	*p*-value for interaction
R1	43 (30–56)	75 (48–100)	32 (5–59)	0.02	0.23
R2	50 (30–70)	68 (40–96)	18 (−14-49)	0.27	
R3	45 (18–72)	65 (28–100)	20 (−17-56)	0.30	
Total	45 (34–56)	68 (53–85)	23 (5–43)	0.01	

R1 = First-year resident (Intern), R2 = Second-year resident (Junior Resident), R3 = Third-year resident (Senior Resident)

Next, we investigated whether adherence varied by level of training. We found that year of training was not associated with adherence after adjusting for elective time and interest in primary care. First year residents were adherent 47 % of the time (95 % CI 36–59), second year residents 51 % of the time (95 % CI 34–68) and third year residents 46 % of the time (95 % CI 13–80), *p* = 0.91 for group difference (not shown in the table). These rates were similar in an unadjusted model.

We then evaluated whether an interest in primary care was associated with adherence. Among residents, 12 % overall were interested in primary care careers and interest in primary care was similar across years. After adjusting for elective time and training year, residents interested in primary care were adherent approximately 64 % of the time (95 % CI 38–90) compared to 46 % among those not interested in primary care (95 % CI 36–56), though this difference was not statistically significant (*p* = 0.22).

Next, we evaluated whether total time spent logged into the EMR varied by rotation assignment and by training year. In an unadjusted model, we found that among residents who logged in at least once per week, average time spent per week did not differ among residents on elective rotations compared to those on required rotations. Those on electives spent an average of 53 min logged in per week (95 % CI 42–65) while those on required rotations spent an average of 55 min (95 % CI 25–86, difference of 1.8 min, *p* = 0.90). This difference did not change after adjusting for training year and interest in primary care (difference of 1.7 min, *p* = 0.90). Of note, on average, since interns see fewer patients per clinic half day and also have fewer clinic half days, interns see approximately 40 % fewer patients than residents over the span of a year. However, time spent logged in also did not vary by year of training. First year residents spent an average of 45 min logged in (95 % CI 33–58), second year residents spent an average of 72 min (95 % CI 42–103) and third year residents spent an average of 46 min (95 % CI 23–69, *p* = 0.91). Results were largely unchanged in a model that included year of training and rotation type (Table 3).

**Table 3:** Time spent logged in to the EMR by training year

	Mean minutes spent logged in (95 % CI)	Unadjusted difference in minutes (95 % CI)	*p*-value	Adjusted difference in minutes^a^ (95 % CI)	*p*-value
R1	45 (33–58)	Ref.	-	Ref.	-
R2	72 (42–103)	27 (−6-60)	0.11	28 (−3-59)	0.07
R3	46 (23–69)	0.83 (−26-27)	0.95	3.1 (−26-33)	0.83

R1 = First-year resident (Intern), R2 = Second-year resident (Junior Resident), R3 = Third-year resident (Senior Resident)

^a^model adjusted for rotation type and interest in primary care

## Discussion

For housestaff to fulfill AAIM’s goal of providing “patient-centered longitudinal care”, it is key for housestaff to have the opportunity to “take ownership” of their patients, completing tasks such as responding to patient e-mails and updating patients on their labs and imaging. To our knowledge, our study is the first to examine housestaff compliance with these “between visit” tasks during core inpatient rotations versus electives, and we demonstrate quantitatively how it is particularly difficult accomplishing these tasks during core inpatient rotations (which comprise the bulk of most residency programs) [2].

Our study presents two key findings: first, we found that during required rotations, rates of adherence to expectations for “between visit” follow up was low, with less than half of residents achieving compliance. During elective months, though, residents were much more likely to log in, with over two thirds of residents achieving compliance. Second, we found that once logged in, residents spent around an hour per week in the EMR, regardless of which kind of rotation they were on.

This pattern suggests that being on an inpatient/required rotation is a significant barrier to engaging in continuity clinic tasks. It also suggests that this barrier may not be, strictly speaking, because of time constraints. After all, once logged in, residents on required rotations were able to spend as much time as their peers on electives attending to outpatient tasks. Rather, it may be that residents on inpatient rotations lack the opportunity to log in (for example if they are at a site with an incompatible computer system) or simply do not have the attention or mental energy to stop and shift tasks in the middle of a busy day on an inpatient service.

Additionally, we found that engaging in outpatient “between visit” responsibilities requires approximately an hour or less per week. These findings are consistent with prior studies which showed that housestaff assigned to an ambulatory block demonstrated an average usage of 1.2 h of after-hours EHR time per week [10]. This again suggests that perhaps it is not the actual time required that prevents residents from completing between-visit tasks, but rather the energy needed to shift attention to a different set of concerns.

Interestingly, we found that year of training did not predict adherence or total time spent logged in. This is somewhat surprising, as we hypothesized that residents further along in their training would have both a greater sense of fidelity toward their primary care panel as well as more patients to attend to (resulting in more time spent logged in), given that R2s and R3s see more patients in clinic than interns do. Among residents in our sample, those interested in primary care, on average, were more likely to be adherent, though this difference was not statistically significant, perhaps because of our small sample size.

This study has several important limitations. First, because our study population was limited to a single clinic, our results may not be generalizable to other programs that may have different infrastructure and expectations for their trainees. Second, electronic audits may under or over estimate time actually spent on between visit tasks. For example, time spent on the phone with patients or other providers may not be reflected in log in time. On the other hand, time spent logged in but idle is still captured. Additionally, we may capture remote log ins that were not for outpatient duties. Our study design assumes that housestaff assigned to an off-site rotation would only login remotely to the main campus EHR system for continuity clinic purposes. However, we cannot exclude that some logins may have been to attend to overdue discharge summaries from beyond our 1 month exclusion period. Importantly, based on these limitations, our study could potentially overestimate compliance with checking outpatient inboxes but could not underestimate compliance. Lastly, our sample was small, with only 41 residents and limited follow up for each. In particular, the number of R3s was small in our sample. Because of this small sample, we may be underpowered to detect some group differences and in general, our estimates had wide confidence intervals.

Furthermore, while it is beyond the scope of our current study, other indicators of performance could be helpful as well, such as patient satisfaction metrics or manual chart review of resident actions (i.e., e-mail responses to patient inquiries or fulfillment of medication refill requests). Such additional indicators of performance could be incorporated into future studies.

For residency programs, the challenge of balancing inpatient and outpatient responsibilities during IM residency is as pressing as ever. Given that trainees in our study who were interested in primary care also found it difficult to comply with outpatient expectations while assigned to inpatient rotations, the expectations of both inpatient and outpatient curricula should continue to be reexamined.

Our results reinforce the need to continue development and study of innovative systems for coverage of “between visit” responsibilities, including shared coverage models among multiple residents and shared coverage models between residents and clinic attendings. In a survey of resident clinic structure nation-wide, nearly two-thirds of clinics (136 of 221) reported either a partial or complete “firm system” in which discrete teams support provision of care [10]. When the primary resident was unavailable, approximately one-third (*n* = 75) had a resident “buddy” designated to see the patient, but the majority of clinics (*n* = 160, 73 %) stated that the patients are seen by any available resident or faculty member. Most of the clinics also had clerical and nursing support with variable support from pharmacy and other ancillary staff [11].

While greater support by RNs or attendings may help in lowering the burden of “between visit” tasks for residents, such support may also detract from the patient’s and resident’s sense of continuity and ownership. Future research should be directed at assessing the impact of new systems on housestaff satisfaction and perception of “patient ownership”, patient satisfaction and compliance with “between visit” tasks. This is critical as it has been shown that the organizational environment of continuity clinic influences learning and that residents rate their clinic experience largely based on the operational issues of their clinic [12].

## Conclusions

Using a novel approach, our research demonstrates that time spent on busy inpatient rotations makes concurrent attention toward crucial “between visit” outpatient responsibilities difficult. Future research focused on comparing different coverage systems may inform discussions regarding the best balance between education and patient care and between inpatient and outpatient responsibilities.

## Ethics approval and consent to participate

Our study’s retrospective data collection and analysis was approved by the Stanford Institutional Review Board. Consent to participate was not deemed necessary.

## Consent for publication

Not applicable.

## Availability of data and materials

We uploaded a spreadsheet of raw data.

## Abbreviations

EHR: electronic health record
IM: Internal Medicine
